# A Systematic Review and Bayesian Network Meta-Analysis Investigating the Effectiveness of Psychological Short-Term Interventions in Inpatient Palliative Care Settings

**DOI:** 10.3390/ijerph19137711

**Published:** 2022-06-23

**Authors:** Reka Schweighoffer, Andrea M. Schumacher, Richard Blaese, Silke Walter, Sandra Eckstein

**Affiliations:** 1 Department for Clinical Research, University of Basel, Missionstrasse 64, 4055 Basel, Switzerland; 2 Department of Psychology, University of Basel, Missionsstasse 62, 4055 Basel, Switzerland; andrea.schumacher@upd.unibe.ch (A.M.S.); r.blaese@unibas.ch (R.B.); 3 Chief Medical and Chief Nursing Office, Department of Practice Development and Research, University Hospital Basel, Hebelstr. 2, 4031 Basel, Switzerland; silke.walter@usb.ch; 4 Department of Palliative Care, University Hospital Basel, Petersgraben 4, 4031 Basel, Switzerland; sandra.eckstein@usb.ch

**Keywords:** palliative care, short-term psychotherapy, inpatient setting, meta-analysis

## Abstract

This paper reviews and summarises the evidence of short-term psychosocial interventions (up to 12 sessions delivered within less than eight weeks) on anxiety, depression, and emotional distress in palliative patients in inpatient settings. We screened publications from the following five databases, Embase, PubMed, PsycINFO, Web of Science, and CINAHL, from their inception to 10 September 2021. The eligible studies included controls receiving standard palliative care, actively treated controls, and wait-list controls. Nine studies met the eligibility criteria and reported the effects of five psychosocial interventions in a total of *N* = 543 patients. We followed PRISMA-guidelines for outcome reporting and the Cochrane Risk of Bias Assessment Tool for assessing study quality. This paper used the network meta-analysis to compare multiple treatments by providing greater statistical power and the cross-validation of observed treatment effects, using the R package BUGSnet. Compared to control groups, the following psychosocial interventions in inpatient settings showed to be superior: life review interventions were the best ranked treatment for improving anxiety and distress, while the top ranked treatment for reducing depression was outlook intervention. The short-term psychosocial interventions investigated in this meta-analysis, especially life review intervention, are feasible and can potentially improve anxiety, depression, and distress in palliative inpatients and should therefore be offered in inpatient settings.

## 1. Background

The need for psychological support is tremendous in patients nearing their end of life. Patients in palliative situations often suffer from depression and anxiety or feelings of hopelessness, demoralisation, loss of control, and loss of quality of life [1,2]. Palliative inpatients (PIP) usually stay in hospital for a short period of time and the condition of PIP often rapidly deteriorates within this short timeframe. Long-term psychological interventions over multiple sessions are therefore difficult to perform, which is why PIP could greatly benefit from brief psychotherapies tailored to their specific needs [3,4]. Contrary to long-term psychotherapies, short-term psychotherapies are typically more goal-oriented and tend to focus on specific challenges that are causing patients the greatest amount of adversity in the present. To date there is no uniform definition or consensus on the length or extent of short-term therapies. According to Hazlett-Stevens, short-term psychotherapies lasted up to 10–20 sessions and were delivered within a timespan of approximately two to four months [5]. Stern et al. and Beck et al. defined brief psychotherapy as an intervention with a predetermined endpoint that usually consisted of 8 to 12 sessions [6,7]. We defined psychological short-term interventions as interventions that include from one to twelve sessions delivered over a time interval of a maximum of eight weeks, which corresponds to suggested timeframes of the cited literature.

Recent meta-analyses demonstrated small to large effects in the reduction of depression, anxiety, and distress in PC patients through cognitive behavioural therapy (CBT) interventions, mindfulness-based interventions, and life-review interventions (LRI, also known as dignity and meaning-centred interventions) [3,4,8,9]. To date, there are several short-term psychological interventions for PIP available, while novel psychosocial interventions to address end-of-life care issues are on the rise [10]. CBT focuses on changing automatic negative thoughts or patterns of behaviour that contribute to and worsen emotional difficulties, depression, and anxiety. Impairing patterns are identified, challenged, and replaced with more objective thoughts and behaviours [7]. There is strong evidence for the efficacy of CBT for various disorders in different patient populations, especially for symptoms of depression and anxiety [11,12]. Mindfulness-based interventions (MBT) consist of various meditation exercises and entail elements that focus on intentionally directing one’s attention to experiences that occur in the present moment [13]. In the past, MBT proved to be especially effective in reducing feelings of stress, pain, anxiety, depression, and restlessness [14].

Life review interventions (LRI), including “dignity therapy”, are brief (usually two sessions) individualised therapies that aim to relieve psycho-emotional and existential distress [15]. LRI offer patients an opportunity to reflect on autobiographical episodes, attitudes, and values that they would like to transmit to others. Typically, the session is recorded, transcribed, and edited, after which a legacy document is created and given to the patient [15]. A similar intervention to LRI and dignity therapy that focuses more on the patient’s outlook on their remaining time, as well as forgiveness, is the “outlook-intervention” invented by Steinhauser and colleagues [16]. LRI approaches are known to be especially effective against existential distress but might also be effective against the anxiety and depression experienced at the end of life [9,15].

Assessment of the extent of the benefits of psychological short-term interventions for PIP is limited by the existence of only a few very heterogenous clinical studies. This is because psychosocial interventions for PIP must meet specific requirements, namely abbreviated session times and therapy durations due to the patients’ acute conditions, as well as minimised questionnaire burden. Moreover, there are high attrition rates due to patients’ unstable conditions. An evaluation of short-term psychological interventions (CBT-based interventions, mindfulness-based interventions, and life-review interventions) for inpatient settings in the form of a network meta-analysis is particularly relevant to gain an initial impression of which short-term therapies are most beneficial for PIP.

Recent literature reviews and meta-analyses typically included patients with a longer life expectancy [3], focused on a single psychotherapy intervention or included other psychosocial interventions such as music or aroma therapy [4], did not distinguish between long-term and short-term psychotherapies [3,5,9], and included mostly ambulant patients and group therapy sessions [3,4,8,9]. To our knowledge, this is the first study to assess short-term psychotherapy with a focus on feasibility in inpatient or stationary palliative settings.

## 2. Methods

Compared to “conventional” pairwise meta-analysis, the method of network meta-analysis allows the simultaneous comparison of multiple (more than two) pairwise short-term psychotherapy treatments, including comparisons that are not directly available in the literature [17]. Moreover, it can do so with studies with small sample sizes and differing sample characteristics and assessment tools [17]. By including a mixed treatment comparisons component, we use the term network meta-analysis to refer to a mixed treatment comparison meta-analysis throughout this manuscript. This study was registered in PROSPERO (ID: CRD42020213019) and conducted in accordance with PRISMA guidelines [18].

### 2.1. Eligibility Criteria

Studies were selected in accordance with the participants, interventions, comparators, outcomes, and study designs (PICOS) framework [19]. The inclusion criteria were as follows:

(1) Participants: PIP of 18 years and older; at least 50% of the study population must be inpatients.
(2) Intervention: psychological short-term interventions (e.g., mindfulness-based interventions, LRI, CBT, and related approaches) administered within eight weeks from baseline to follow-up assessment; number of sessions may range from one to twelve.
(3) Comparison groups: control groups, e.g., standard care, no treatment, waitlist control, active attention control.
(4) Outcomes: anxiety, depression, and distress.
(5) Study designs: randomised controlled trial (RCT), clinical controlled trial, or waitlist-controlled trial.

This systematic review and meta-analysis focused on psychological interventions that are feasible for PIP. We defined psychological short-term interventions as interventions that include from one to twelve sessions delivered over a time interval of a maximum of eight weeks. We also included studies that reported outcomes at a first assessment within the eight-week time interval.

The exclusion criteria were as follows:

(1) Studies of complementary therapies such as physical (e.g., yoga, physiotherapy), music, art, or aromatherapy as well as group, partner, or caregiver-delivered therapies.
(2) Interventions delivered online and solely by telephone.
(3) Studies written in a language other than English or German.
(4) Grey literature, conference proceedings, abstracts, posters, editorials, protocols, unavailable full texts, or studies that report only qualitative results.
(5) Studies with insufficient information on outcome data or inclusion criteria (studies comparing only two equal interventions with no additional control condition were excluded).

### 2.2. Search Strategy

A systematic literature search was conducted in the following five databases: Embase, PubMed, PsycINFO, Web of Science, and CINAHL (see Appendix A for search terms used). Records were considered from the inception of the databases to 10th of September 2021. In addition, reference lists of previously published systematic reviews were manually screened.

### 2.3. Study Selection

All records obtained from the databases and hand search were exported to Excel and screened by title by two reviewers (AS and RS). At the initial screening stage, the authors read the titles and abstracts of the candidate studies and decided to include or exclude each study from the review. Any duplicates were removed. Relevant full texts were examined according to inclusion and exclusion criteria. In cases where it was unclear from the title or abstract whether a paper was relevant, a copy of the full text of the study was sought for consideration. Any uncertainty was discussed between the two reviewers (AS and RS) and, if consensus could not be reached, another author assisted with final decision-making (SE or SW). Corresponding authors of eligible studies were contacted if information on inclusion and exclusion criteria could not be obtained from the full text.

### 2.4. Analysis Method

The network meta-analysis (NMA) was performed using the BUGSnet package on R [20]. All NMA models were based on a Bayesian approach through the Markov Chain Monte Carlo (MCMC) simulation [21]. The parameters assessed in the NMA models were treatment effect compared to other treatment arms and the likelihood function was dependent on the outcome. Continuous data were pooled as mean difference (MD) with posterior median and 95% credible intervals displayed for the outcomes HADS total, depression, anxiety, and distress. Surface Under the Cumulative Ranking Curve (SUCRA) analysis was performed to rank treatment arms according to their efficacy. SUCRA plot and score are presented in the results. Model selection and goodness-of-fit were evaluated through deviance information criteria (DIC). Adequacy of the model fit was assessed through a comparison of the residual deviance of the models, where a close match between both models was considered an adequate fit. The Brooks–Gelman–Rubin diagnostic was employed to assess the convergence through visual inspection, with a potential scale reduction factor (PSRF) value of <1.05 considered as an indication that the simulation is valid.

## 3. Results

### 3.1. Result of the Literature Search

**Figure 1:**
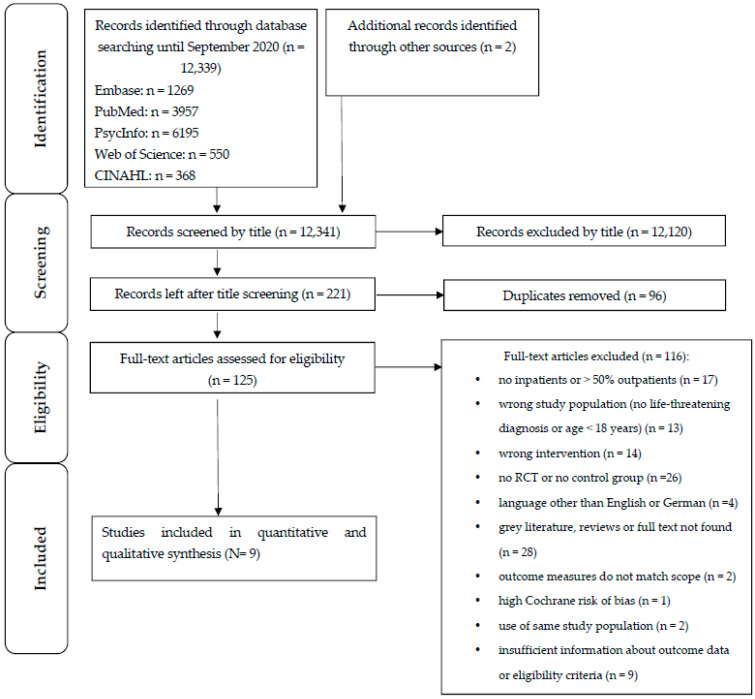
Flow diagram of study selection.

The literature search and study selection are shown in Figure 1. A total of 12,339 records were identified in five databases. In addition, two records were added that were found by hand searching, yielding a total of 12,341 records. Of these, 12,120 were excluded after title screening, leaving 221 records. After removing 96 duplicates, 125 full texts were screened. As Figure 1 shows, 116 full texts were excluded, as they did not fulfil the eligibility criteria, and nine studies were excluded because the authors did not respond to our request for more information about the eligibility criteria or outcomes. Nine studies were included in the final analysis.

### 3.2. Data Collection Process

The detailed information of the selected studies is summarised in Table 2. The extracted sample sizes, means, and standard deviations of the outcomes were assessed by two reviewers (AS and RS) under the Appendix A.

### 3.3. Outcome Measures

#### 3.3.1. Depression

Seven of the included studies [22,23,24,25,26,27,28] assessed depressive symptoms with the depression subscale of the Hospital Anxiety and Depression Scale (HADS; Ref. [29]). Savard et al. [26] additionally used the Beck Depression Inventory (BDI; Ref. [30]), Steinhauser et al. [16] used the Center for Epidemiological Studies Depression Scale (CES-D; Ref. [31]), and Rodin et al. [32] used the Beck Depression Inventory-II (BDI-II; Ref. [33]) to assess depressive symptoms.

#### 3.3.2. Anxiety

All included studies that assessed anxiety symptoms used the anxiety subscale of the HADS [29] except for Steinhauser et al. [16], who used the Profile of Mood States (POMS, Ref. [34]).

#### 3.3.3. Distress

Three of the included studies [22,27,28] assessed distress using a single-item rating scale.

### 3.4. Risk of Bias Assesment

The authors RS and AS assessed and independently rated the quality of the studies using the Cochrane Risk of Bias Assessment Tool [35]. The tool comprises seven criteria (see Table 2). It is generally known that the quality criteria “blinding of participants and personnel” is not conferrable in studies that deliver psychological interventions to patients [36]. However, for the sake of completeness we did not exclude this criterion and rated studies that did not fulfil this criterion as “high” (see Table 2). If information was missing or incomplete, the field was judged as “unclear”.

Table 1 presents the results of the risk of bias assessment. Seven studies [22,23,24,25,26,28,32] fulfilled five out of seven criteria with “low bias”. One study [37] had to be excluded, as all criteria were rated as “high risk” or “unclear”.

## 4. Study Characteristics

The study characteristics of the final nine studies are listed in Table 2. Most were conducted in North America (*n* = 3; Refs. [16,26,32]), Asia (*n* = 3; Refs. [22,25,27]), Europe (*n* = 2; Refs. [24,28]), and South America (*n* = 1; Ref. [23]). All studies were published in peer-reviewed journals between 2006 and 2020. All were randomised-controlled trials, apart from one randomised crossover trial [28]. Two studies applied a three-arm RCT design [16,23]; only two of these three arms (targeted intervention and control condition) were included in the analysis. The total sample size of all studies used for the meta-analysis consisted of *N* = 543 at baseline. The patients’ age ranged between 19 to 96 years and the proportion of female patients varied from 38.10% to 100%. The patients’ remaining life expectancy varied between 1 to 24 months. Inclusion and accrual rates across the included studies were described as generally low (with approximately 5–20%) and most studies reported high to very high attrition rates of up to 55% due to worsened medical conditions throughout the study course.

Patients in six studies [22,24,26,27,28,32] were recruited exclusively in inpatient PC settings, while three studies [16,23,25] recruited in both inpatient and outpatient settings. However, these three studies fulfilled our eligibility criteria of at least 50% of inpatients. All patients suffered from life-limiting diseases. Five studies focused on patients with a terminal cancer diagnosis (*n* = 3 of all cancer types [22,23,27] and *n* = 2 [26,32] of homogenous cancer diagnoses) and four studies included a minority of patients with other life-limiting diseases [16,24,25,28]. The patients’ life expectancy varied between 1 to 24 months [22,27,28,32]. Studies investigated CBT-based interventions (*n* = 2), short-term LRIs (*n* =3), mindfulness-based interventions (*n* = 3), outlook intervention (*n* = 1), and emotion- and symptom-focused engagement (*n* = 1; an intervention based on trauma-focused therapy and supportive psychotherapy). Five studies reported outcomes on the Hospital Anxiety and Depression Scale (HADS) total, which reports aggregated data on anxiety and depression in a hospital setting. Further outcomes that were assessed by the studies were anxiety and depression, as well as distress (assessed by distress scales and thermometers). For an overview of the different assessment tools that were used to measure the outcomes, see Table 2. To allow for comparison between the different outcome measures, we applied a Bayesian meta-analysis approach.

### 4.1. Anxiety and Depression (as Measured by the HADS Total)

**Figure 2:**
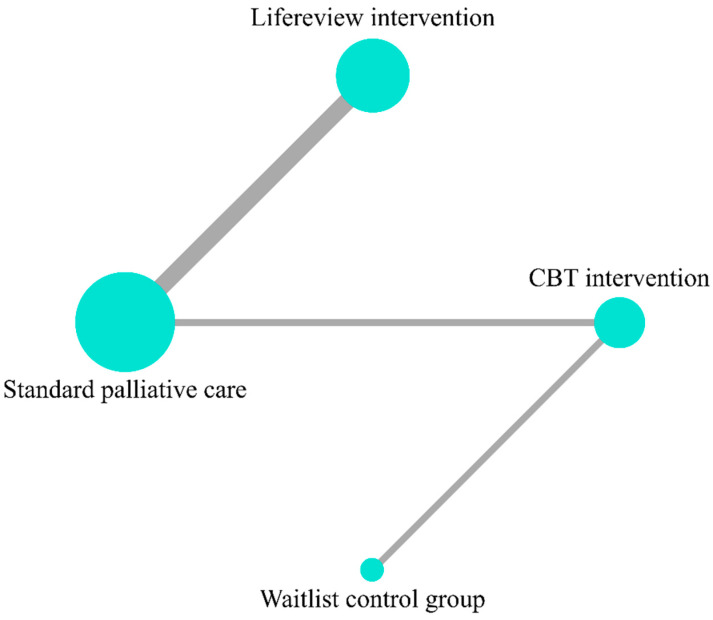
Network plot for outcome of HADS total.

**Figure 3:**
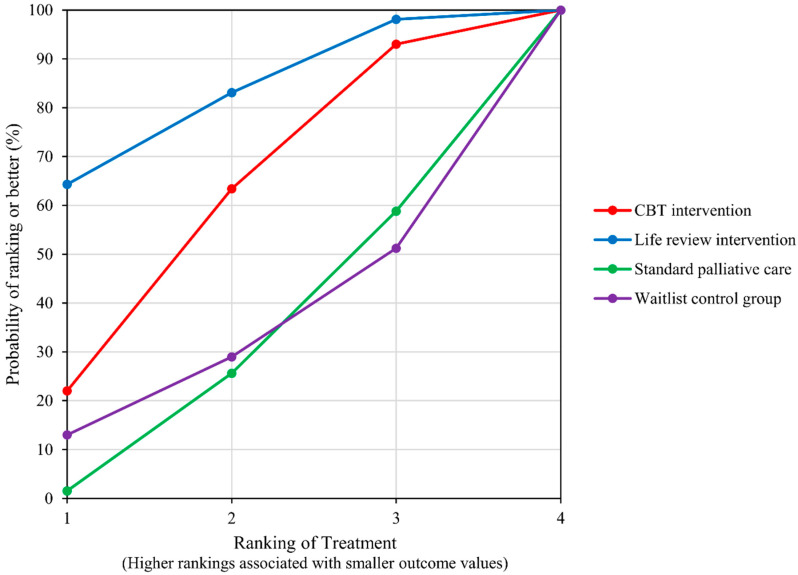
SUCRA Plot for outcome of HADS total.

Five studies [22,23,24,25,26] provide data on the HADS total score and include five groups for comparison (control groups included) (Figure 2, Network plot). LRI was the best ranked treatment for improving HADS total score (SUCRA = 81.6), followed by CBT-based psychosocial intervention (SUCRA = 59.3) and waitlist control group (SUCRA = 30.7). Standard PC was the lowest ranked treatment for improving HADS total (SUCRA = 28.3); (Figure 3, SUCRA plot). In pairwise direct comparison, there was no significant difference between the network treatment arms.

### 4.2. Depression

**Figure 4:**
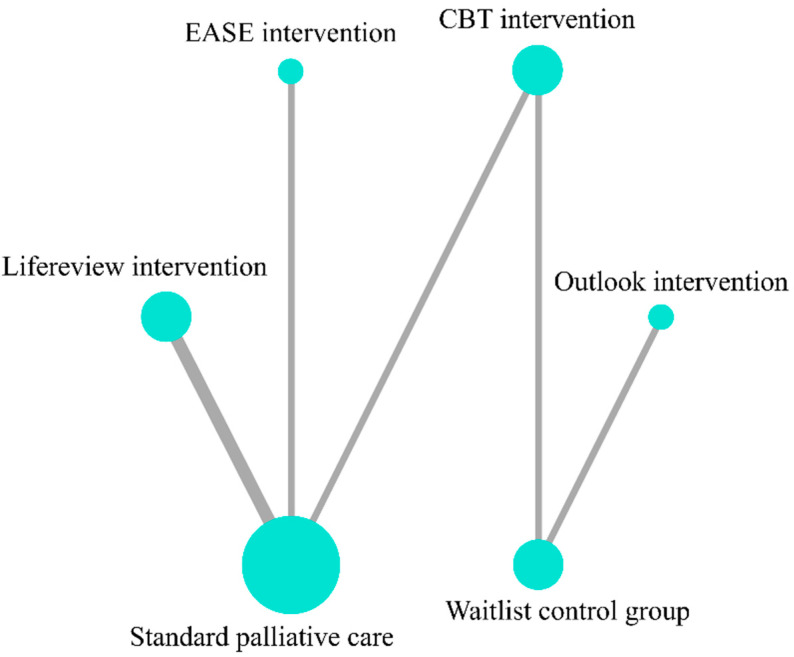
Network plot for outcome of Depression.

**Figure 5:**
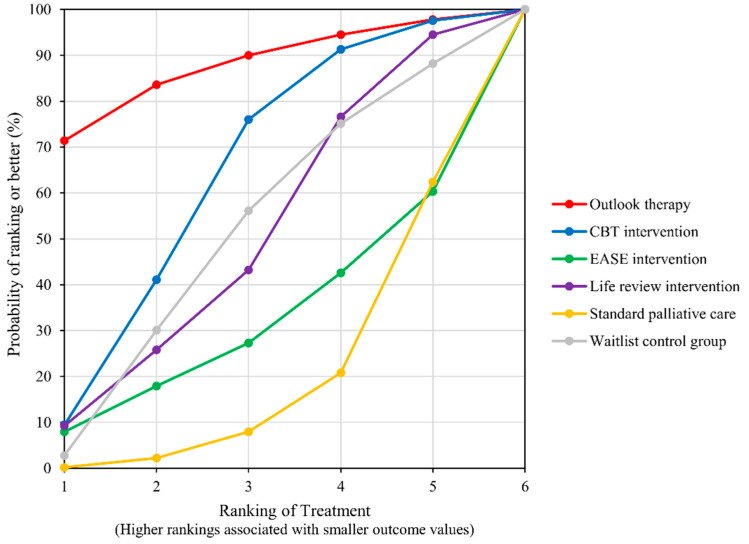
SUCRA plot for outcome of Depression.

Data on depression were provided by six studies [16,23,24,25,26,32] that included seven groups for comparison (Figure 4, Network plot). The top ranked treatment for reducing depression was outlook intervention (SUCRA = 87.1), followed by CBT-based psychosocial intervention (SUCRA = 63.1) and control group (SUCRA = 50.8). LRI (SUCRA = 49.6), Emotion and Symptom-focused Engagement intervention (EASE) (SUCRA = 30.8), and standard PC (SUCRA = 18.7) were the bottom ranked treatment options (Figure 5, SUCRA plot). No significant difference among the network treatment arms was detected via pairwise comparison.

### 4.3. Anxiety

**Figure 6:**
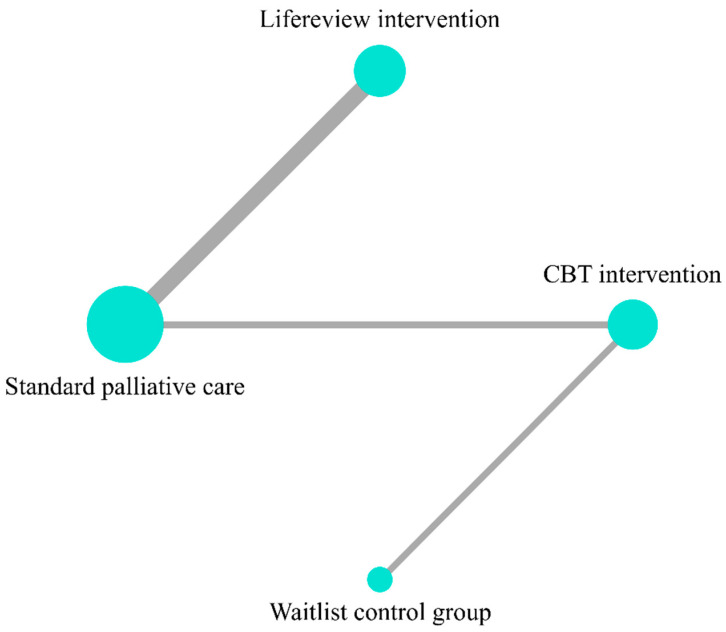
Network plot for outcome of Anxiety.

**Figure 7:**
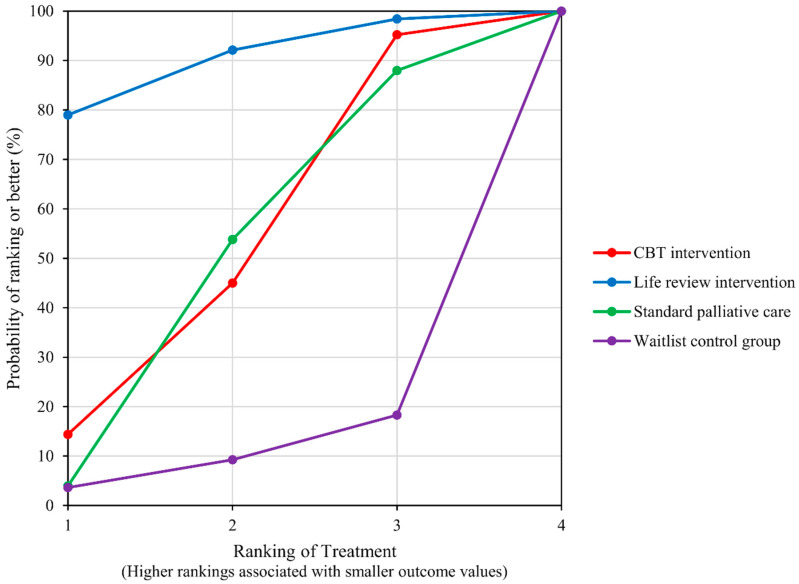
SUCRA plot for outcome of Anxiety.

Data on anxiety was reported by five studies [16,23,24,25,26] that included five groups for comparison (Figure 6, Network plot). LRI was the best ranked treatment for improving anxiety (SUCRA = 89.7), followed by CBT-based psychosocial intervention (SUCRA = 51.4) and standard PC (SUCRA = 48.5). Waitlist control was the worst ranked group (SUCRA = 10.3); (Figure 7, SUCRA plot). No significant difference among the network treatment arms was detected via pairwise comparison.

### 4.4. Distress

**Figure 8:**
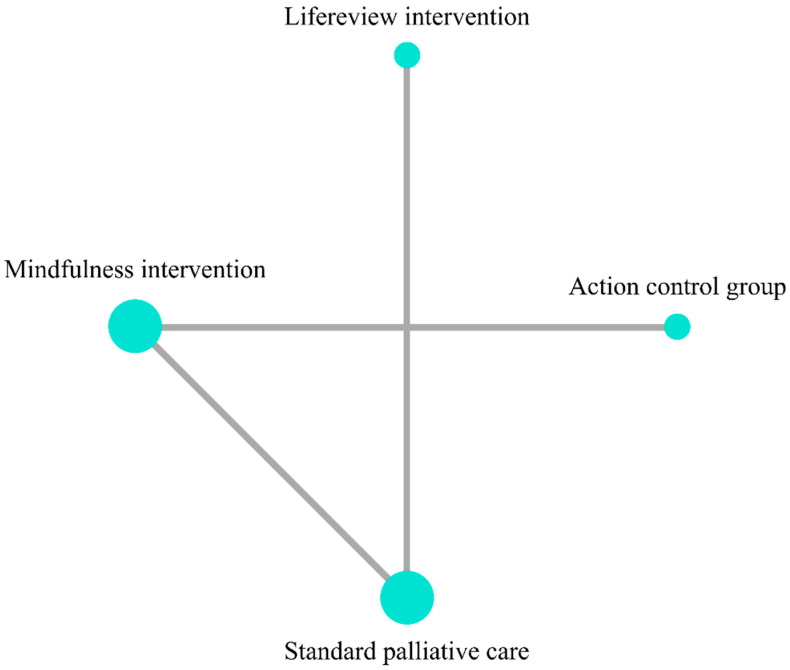
Network plot for outcome of Distress.

**Figure 9:**
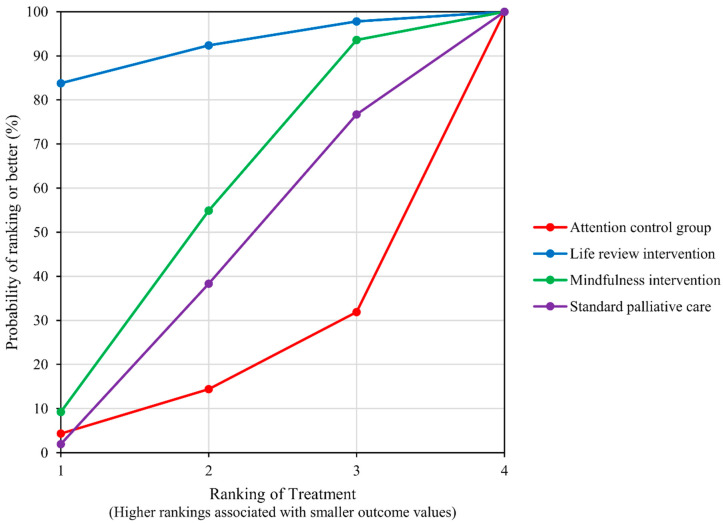
SUCRA plot for outcome of Distress.

Three studies [22,27,28] provided data on distress (Figure 8, Network plot). LRI was the best ranked treatment (SUCRA = 91.3), followed by mindfulness-based intervention (SUCRA = 52.7) and standard PC (SUCRA = 39), while the attention control group was the lowest ranked group for relieving distress (SUCRA = 17); (Figure 9, SUCRA plot). No significant difference among the network treatment arms was detected via pairwise comparison.

To assess the convergence, a Gelman--Rubin--Brooks plot was constructed. It showed that the Potential Scale Reduction Factor (PSRF) was below 1.05, indicating that the simulations performed are valid (Supplementary file S1. Gelman convergence plot). The deviance report showed the contribution of each study arm to the residual deviance, where all points were around 1, close to the equality line, and there were no extreme values, indicating that no study had a high residual deviance (Supplementary file S2. Deviance plot). As for the contour plot, no leverage values were outside the contour of 3 in the REM, indicating that no study is considered poorly fitting (Supplementary file S3. Leverage plot).

## 5. Discussion

To provide an overview of the impact of short-term psychotherapies performed by trained healthcare personnel for PIP, we conducted a systematic Bayesian network meta-analysis (*N* = 543). We defined a “short-term intervention” as one that would rely on a maximum of twelve sessions and a treatment duration of less than eight weeks. The included studies investigated CBT-based interventions (*n* = 2), short-term LRI (*n* =3), mindfulness-based interventions (*n* = 3), outlook intervention (*n* = 1), and emotion- and symptom-focused engagement (*n* = 1). Overall, we found a positive effect of short-term psychotherapies on patient outcomes of anxiety, depression, and distress. Furthermore, we demonstrated that they are feasible in inpatient settings.

Considering the results of our meta-analysis, we found that LRI was the best ranked treatment for improving overall HADS scores and for improving anxiety and distress in PIP, while the top ranked treatment for reducing depression was outlook intervention that contains components of LRI. That LRI /dignity therapy was the highest rated treatment overall is consistent with previous review findings that imply that LRI interventions are effective against anxiety and depression in PC patients [38] and improve overall quality of life [39,40]. CBT-based therapies appeared to be the second most helpful intervention for anxiety and depression, while mindfulness-based interventions turned out to be the second-best treatment option for distress. This finding is in accordance with a previous meta-analysis [41] that demonstrated that CBT-based interventions, among others, were effective in reducing symptoms of depression and anxiety in adults suffering from serious illness. In our meta-analysis, the “standard PC” control group achieved good results for anxiety and depression as compared to no care. This was expected according to recent literature, as standard PC usually entails some form of psychosocial support for patients and their relatives [9].

For symptoms of distress, mindfulness-based interventions proved to be a good treatment option. This finding is in line with a recent review and meta-analysis which found that mindfulness-based interventions appeared efficacious in reducing psychological distress and other symptoms in cancer patients and survivors [42]. 

Our results provide compelling evidence that life-review intervention is particularly helpful in reducing anxiety and depression in PC inpatients. That LRI can be delivered in as few as two sessions is crucial for inpatient settings, where the condition of palliative patients can deteriorate rapidly, and hospitalisation is of short duration [38]. Furthermore, LRI therapy is particularly cost- and resource-efficient, with relatives also benefiting from the documents the patients leave to them [39,40,43]. We therefore recommend that specialised inpatient palliative care settings invest in the LRI training of healthcare professionals. Ultimately, however, the choice of therapy must always consider the individual preferences of the patient and the patient’s needs.

There are some limitations that should be considered for the interpretation of our results. First, two studies that otherwise fulfilled all inclusion criteria could not be included in our meta-analyses due to insufficient data that would have limited the generalisability of our results. Second, risk of bias was high in all studies. This may be due to the use of the Cochrane tool for assessment, which was originally developed for general randomised controlled trials. Even though we used a version that was adjusted for psychotherapy research [36], all included studies performed rather poorly in the assessment. This phenomenon seems to be the norm and was shown in other meta-analyses with similar patient populations [4], as patient populations nearing their end-of-life are particularly vulnerable and clinical trials are usually not feasible under the highest quality standards.

Another critical issue refers to the small number of studies included, especially in the meta-analysis on distress (*n* = 3). Some of the included studies had very small sample sizes. To anticipate an underpowered analysis, we specifically applied the method of network meta-analysis. However, future studies in this field with higher sample sizes are warranted. According to Do Carmo et al. [23], future studies to be conducted with this population group need to revise the eligibility criteria and make them less restrictive, so that higher participation rates can be achieved. However, as previously discussed, this will further increase the study’s risk of bias.

Future reviews and meta-analyses are encouraged to investigate the categorisation of effects in different treatment phases of palliative care, which would enable a more precise overview of the patients’ needs in each phase and benefit therapeutic intervention design. This was conducted by Warth et al. [4], who investigated the effect of music therapy in different stages of cancer patients.

Regarding moderating factors, interesting factors such as pre-existing psychological conditions or experiences with/preferences for psychotherapy could not be considered, as they were rarely assessed in the included studies. Moreover, this meta-analysis did not consider which profession delivered the intervention or the training of the therapists. Future research should consider examining these aspects as potential modulating variables. Another possible influence which studies often do not report is PIPs’ intake of psychopharmaceuticals and pain medication, especially opioids. PIPs often receive high doses of pain medication and a wide range of psychopharmaceuticals to ease their physical and emotional discomfort. These are control variables of high interest, since their impact is possibly confounded with the effect of psychotherapeutic interventions on anxiety, distress, and depression. With respect to future research, studies on short-term psychotherapies in inpatient PC settings are encouraged to reduce the risk of bias, for example through assessing confounding variables, publishing primary and secondary outcomes in a respective study protocol, and documenting longer lasting effects of the applied interventions. As this paper was focused on mostly inpatient samples (patients of papers [22,24,26,27,28,32] were recruited exclusively in inpatient PC settings), it is unclear to what extent the results are generalisable to an ambulatory or home care setting. Three of the included papers included a smaller proportion of ambulant patients, or patients who were referred back and forth between outpatient and inpatient settings. Swift referrals from one setting to the other are very common, as often inpatient facilities can keep patients only for a limited time due to cost and capacity constraints. However, with the inclusion criterion of at least 50% inpatients per paper, we prove that the investigated short-term psychotherapies are indeed feasible in the inpatient setting. Future research on the effect of different psychotherapy treatments exclusively on PIP is highly warranted.

With this analysis we open a new discourse which is yet missing in terms of the effectiveness of short-term psychological therapies specifically offered to PIP. To date, short-term PC interventions applied in a clinical setting are heterogeneous in content and delivery. Furthermore, there is no best practice for treatment for anxiety, depression, and distress in PIP. However, this meta-analysis demonstrates that different short-term psychological interventions for PIP are feasible, are beneficial, and that there is a need to make short-term psychotherapies in hospitals and hospices available to palliative patients.

## 6. Conclusions

The findings of this meta-analysis show that it is feasible and beneficial to grant PIP access to psychological short-term interventions. LRIs consisting of only two to four sessions seem to have great potential to help PC patients cope with anxiety and depression. However, results should be interpreted with caution due to the limited number of randomised controlled trials and associated methodological weaknesses. Further rigorously designed randomised controlled trials with psychological short-term interventions are warranted in inpatient palliative care settings.

## Figures and Tables

**Table 1:** Risk of bias assessment of included studies.

Study	RAND	ALLO	BLPP	BLOA	INCDAT	SELREP	OTBI
Steinhauser et al., 2008 [16]	?	?	HIGH	LOW	LOW	LOW	LOW
Ando et al., 2010 [22]	LOW	LOW	HIGH	LOW	?	LOW	LOW
Do Carmo et al., 2017 [23]	LOW	LOW	HIGH	LOW	?	LOW	LOW
Juliao et al., 2014 [24]	LOW	LOW	HIGH	LOW	?	LOW	LOW
Kwan et al., 2019 [25]	LOW	LOW	LOW	LOW	?	LOW	HIGH
Savard et al., 2006 [26]	LOW	LOW	HIGH	LOW	HIGH	LOW	LOW
NG et al., 2016 [27]	?	?	HIGH	?	?	LOW	LOW
Warth et al., 2020 [28]	LOW	LOW	HIGH	HIGH	LOW	LOW	LOW
Rodin et al., 2019 [32]	LOW	LOW	HIGH	?	LOW	LOW	LOW

*Note*. RAND: random sequence generation; ALLO: allocation concealment; BLPP: blinding of participants and personnel; BLOA: blinding of outcome assessment; INCDAT: incomplete data; SELREP: selective reporting; OTBI: other bias. HIGH: high bias of reporting; LOW: low bias of reporting; ?: no information provided.

**Table 2:** Overview of included studies.

Study	Design	Country of First Author	Intervention	Control Group	Sample Size at Baseline	Gender (m/f)	Outcomes	Study Characteristics: -Length to Follow up (FU) after Baseline Assessment; -Number of Sessions; -Duration of Sessions.
Juliao et al., 2014 [22]	RCT	Portugal	LRI	Standard PC	*N* = 80	37 m (46.25%), 43 f (53.75%)	HADS total HADS Depression HADS Anxiety	-FU on day 4 -Two intervention sessions between day 1 and day 4 -Each session lasting for 30–60 min
Ando et al., 2010 [20]	RCT	Japan	LRI	Standard PC	*N* = 68	32 m (47.01%), 36 f (52.94%)	HADS total Distress thermometer	-FU after 1 week -Two sessions within one week -Each session lasting for 30–60 min
Kwan et al., 2019 [23]	RCT	Hong Kong	LRI	Standard PC	*N* = 109	62 m (56.9%), 47 f (43,1%)	HADS total HADS Depression HADS Anxiety	-FU after 8 days -Two sessions within 8 days -Each session lasting for 45 min
Do Carmo et al., 2017 [21]	RCT	Brazil	CBT intervention	Standard PC	*N* = 63	22 m (34.92%), 41 f (65.08%)	HADS total HADS Depression HADS Anxiety	-FU on day 45 (after 6.5 weeks intervention)-Up to 5 to 7 sessions in a week for 6.5 weeks -Each session lasting 45 to 90 min
Savard et al., 2006 [24]	RCT	Canada	CBT intervention	Waitlist control group	*N* = 37	100% f	HADS total HADS Depression BDI HADS Anxiety	-FU after 8 weeks -8 weekly sessions across 8 weeks -Each session lasting 60 to90 min
Steinhauser et al., 2008 [16]	RCT	USA	Outlook intervention	Attention control group	*N* = 42	19 f (46%), 23 m (54%)	Depression CESD Anxiety POMS	-FU after 3 weeks -1 weekly session for 3 weeks (3 sessions in total) -Each session lasting 45 to 60 min
Rodin et al., 2019 [30]	RCT	Canada	Emotion and Symptom-focused Engagement intervention (EASE)	Standard PC	*N* = 42	26 m (61.90%), 16 f (38.10%)	BDI-II	-FU after 4 weeks -8 to 12 sessions for 4 weeks -Each session lasting 30 to 60 min
Warth et al., 2020 [26]	Randomized crossover trial	Germany	Mindfulness intervention	Standard PC	*N* = 42	13 m (31%), 29 f (69%)	Distress VAS	-FU after 20 min of the intervention on the same day -1 session -Session lasting 20 min
Ng et al., 2016 [25]	RCT	Malaysia	Mindfulness intervention	Attention control group	*N* = 60	29 m (48.30%), 31 f (51.70%)	One-item numeric distress scale	-FU took place 5 min after the end of intervention -1 session -Session lasting 5 min

*Note.* LRI = Life review intervention; PC = Palliative care; RCT = Randomised control trial; CBT = Cognitive behavioural therapy; HADS = Hospital Anxiety and Depression Scale; BDI = Beck Depression Inventory; BDI-II = Beck Depression Inventory II; POMS = Profile of Mood States Anxiety Subscale; CESD = Center for Epidemiological Study of Depression Scale; VAS = Visual analogue scale.

**Scheme A1:**
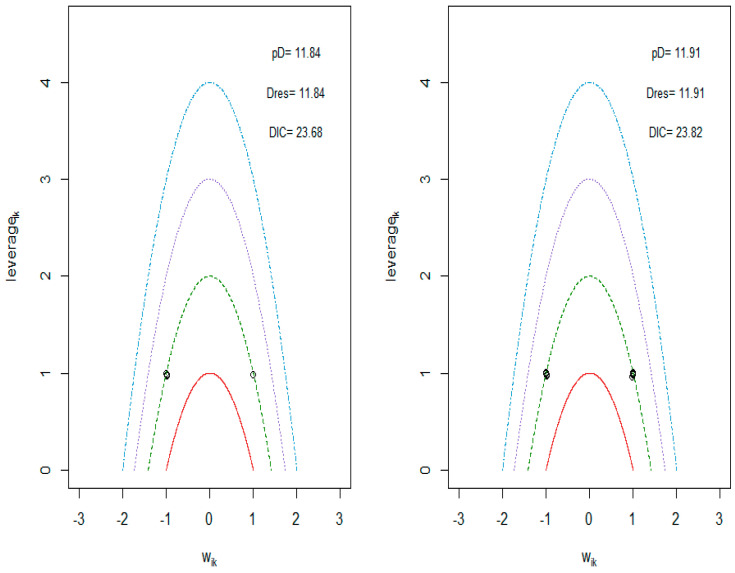
Gelman convergence plot.

**Scheme A2:**
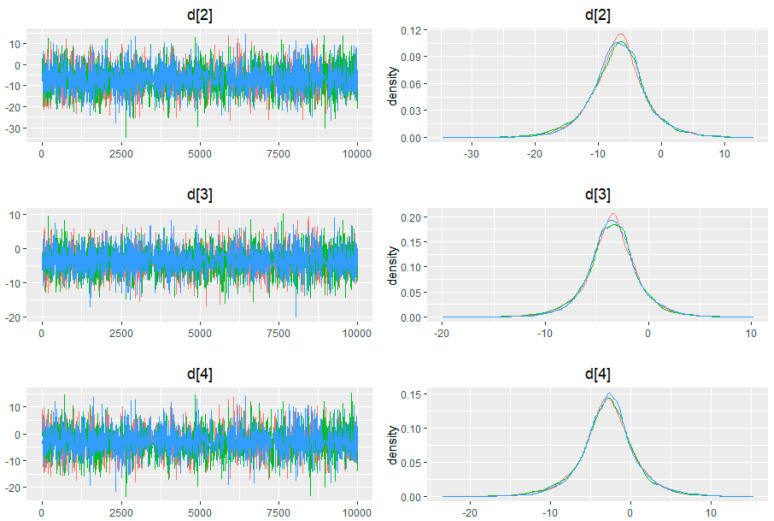
Deviance plot.

**Scheme A3:**
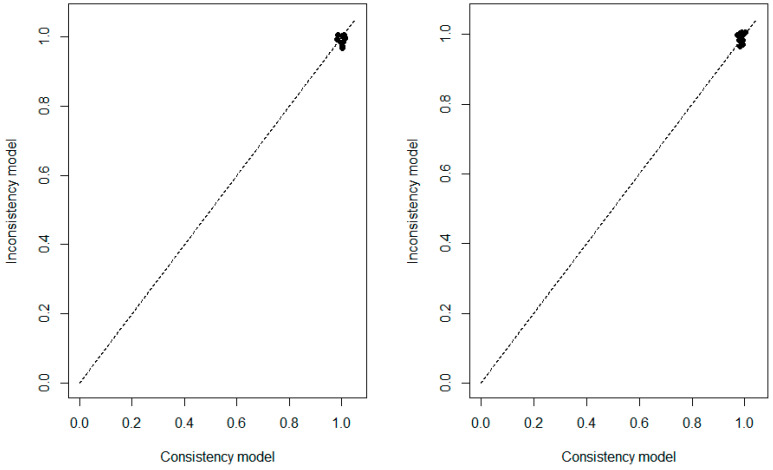
Leverage plot.

## Data Availability

See “Supplementary Materials”. Further data can also be obtained from the corresponding author upon request.

## Supplementary Materials

The following supporting information can be downloaded at: https://www.mdpi.com/article/10.3390/ijerph19137711/s1.

## Appendix A

The following search terms were applied within each data-base: (palliative OR end-of-life OR terminal OR hospice OR terminally ill OR terminal cancer) AND (psychosocial OR psychotherap* OR mind-body OR meaning-centred OR meditation OR cognitive-behavi* OR dignity therapy OR DT OR life review OR psychologic* OR mindful OR CBT OR cognitive behavioural therapy OR individualised meaning-centred psychotherapy OR IMCP OR “managing cancer and living meaningfully” OR CALM OR forgiveness therapy) AND (random* OR RCT OR controlled trial OR CCT OR clinical trial) AND (depression OR depress* OR quality of life OR QOL OR distress OR well-being OR anxiety OR anxi*) AND (inpatient OR hospice OR hospital OR stationary OR nursing home).
