# Supplementary material for: A Systematic Review and Bayesian Network Meta-Analysis Investigating the Effectiveness of Psychological Short-Term Interventions in Inpatient Palliative Care Settings
Source: Int J Environ Res Public Health. 2022 Jun 23;19(13):7711. doi: 10.3390/ijerph19137711 (PMC9265936; doi:10.3390/ijerph19137711)
Supplement: Supplementary file 1 [file ijerph-19-07711-s001.zip › ijerph-1747627-supplementary.pdf]

Statistical values

4 outcome measures = 4 Tables

1. HADS total scale (Hospital Anxiety and Depression Scale)

2. Depression (HADS Depression dimension, BDI I and BDI II)

3. Anxiety (HADS-Anxiety, Anxiety POMS)

4. Distress (VAS-Scales from 0-7 and 0-10)

Table S1. HADS total (composed of HADS Anxiety and Depression) = 6 studies

	BASELINE Intervention group			BASELINE Control group			POST Intervention group			POST Control group		
**Study**	**N**	**Mean**	**SD**	**N**	**Mean**	**SD**	**N**	**Mean**	**SD**	**N**	**Mean**	**SD**
Juliao et al. 2014	HADS Anxiety: 39<br><br>HADS Depression : 39	9.67<br><br>12.87	4.77<br><br>4.79	HADS Anxiety: 41<br><br>HADS Depression: 41	8.88<br><br>13.20	4.36<br><br>3.91	HADS Anxiety: 31<br><br>HADS Depression: 31	5.17<br><br>9.76	0<br><br>3.45	HADS Anxiety: 37<br><br>HADS Depression: 37	8.89<br><br>13.68	3.58<br><br>4.27
Ando et al. 2010	HADS total: 38	17.01	5.6	HADS total: 39	20.1	8.5	HADS total : 34	10.3	3.2	HADS total : 34	21.2	8.3
Kwan et al. 2019	HADS total: 54	11.83	6.68	HADS : 55	12.38	5.82	HADS total: 49	10.73	6.91	HADS total: 40	12.18	6.44

Do Carmo et al. 2017	HADS Anxiety : 19<br><br>HADS Depression : 19	5.79<br><br>5.11	3.92<br><br>4.51	HADS Anxiety: 22<br><br>HADS Depression: 22	6.27<br><br>7.18	4.84<br><br>4.92	HADS Anxiety: 18<br><br>HADS Depression: 17	7.35<br><br>5.76	3.09<br><br>3.12	HADS Anxiety: 19<br><br>HADS Depression: 19	7.16<br><br>9.29	4.37<br><br>3.73
Greer et al. 2012	HADS Anxiety: 20<br><br>HADS Depression : 20	8.79<br><br>9.11	4.64<br><br>5.83	HADS Anxiety: 20<br><br>HADS Depression: 20;	7.93<br><br>7.73	4.45<br><br>3.80	*	*	*	*	*	*
Savard et al. 2006	HADS Anxiety: 21<br><br>HADS Depression : 21	10.95<br><br>9.42	3.57<br><br>2.43	HADS Anxiety: 16<br><br>HADS Depression: 16	12.36<br><br>8.87	3. 80<br><br>2.6	HADS Anxiety: 15<br><br>HADS Depression: 15	6.23<br><br>5.19	3.49<br><br>2.44	HADS Anxiety: 13<br><br>HADS Depression: 13	9.51<br><br>5.83	3.57<br><br>2.45

Table S2. Depression (composed of HADS Depression, Depression CESD, BDI & BDI II) = 7 studies, 8 measures (see Savard et al.)

	BASELINE Intervention group			BASELINE Control group			POST Intervention group			POST Control group		
**Study**	**N**	**Mean**	**SD**	**N**	**Mean**	**SD**	**N**	**Mean**	**SD**	**N**	**Mean**	**SD**
Juliao et al. 2014	HADS Depression : 39	12.87	4.79	HADS Depression: 41	13.20	3.91	HADS Depression: 31	9.76	3.45	HADS Depression: 37	13.68	4.27
Kwan et al. 2019	HADS Depression : 54	9.17	4.52	HADS Depression: 55	8.98	4.87	HADS Depression : 49	8.31	5.01	HADS Depression: 40	8.63	4.94
Do Carmo et al. 2017	HADS Depression : 19	5.11	4.51	HADS Depression: 22	7.18	4.92	HADS Depression: 17	5.76	3.12	HADS Depression: 19	9.29	3.73
Greer et al. 2012	HADS Depression : 20	9.11	5.83	HADS Depression: 20	7.73	3.80	*	*	*	*	*	*
Steinhauser et al. 2008	Depression CESD: 26	11.8	4.6	Depression CESD: 26	10.7	5.1	Depression CESD: 12	8.3	3.3	Depression CESD: 18	12.3	6.0
Rodin et al. 2019	BDI II: 22	10.25	8.11	BDI II: 20	13.20	8.18	BDI II: 14	10.80	7.00	BDI II: N=18	11.19	7.76
Savard et al. 2006	HADS Depression : 21	9.42	2.43	HADS Depression: 16	8.87	2.60	HADS Depression: 15	5.19	2.44	HADS Depression: 13	5.83	2.45

	BDI: 21	21.13	5.41	BDI: 16	20.4	5.76	BDI: 15	11.52	5.42	BDI: 13	15.93	5.52

Table S3. Anxiety (composed of HADS Anxiety (4), Anxiety POMS(1))= 5 studies

	BASELINE Intervention group			BASELINE Control group			POST Intervention group			POST Control group		
**Study**	**N**	**Mean**	**SD**	**N**	**Mean**	**SD**	**N**	**Mean**	**SD**	**N**	**Mean**	**SD**
Juliao et al. 2014	HADS Anxiety: 39	9.67	4.77	HADS Anxiety: 41	8.88	4.36	HADS Anxiety: 31	5.17	0	HADS Anxiety: 37	8.89	3.58
Kwan et al. 2019	HADS Anxiety : 54	2.67	3.12	HADS Anxiety: 55	3.40	3.12	HADS Anxiety: 49	2.42	3.29	HADS Anxiety: 40	3.55	3.69
Do Carmo et al. 2017	HADS Anxiety: 19	5.79	3.92	HADS Anxiety: 22	6.27	4.84	HADS Anxiety: 18	7.35	3.09	HADS Anxiety: 19	7.16	4.37
Greer et al. 2012	HADS Anxiety: 20	8.79	4.64	HADS Anxiety: 20	7.93	4.45	*	*	*	*	*	*
Savard et al., 2006	HADS Anxiety: 21	10.95	3.57	HADS Anxiety: 16	12.36	3.80	HADS Anxiety: 15	6.23	3.49	HADS Anxiety: 13	9.51	3.57

Steinhauser et al. 2008	Anxiety POMS: 26	6.4	4.7	Anxiety POMS: 26	4.4	4.3	Anxiety POMS: 12	4.1	3.5	Anxiety POMS: 18	5.8	5.3

Table S4. Distress VAS= 3 studies

	BASELINE Intervention group			BASELINE Control group			POST Intervention group			POST Control group		
**Study**	**N**	**Mean**	**SD**	**N**	**Mean**	**SD**	**N**	**Mean**	**SD**	**N**	**Mean**	**SD**
Ando et al. 2010<br><br>**VAS from 0-6**	Distress VAS : 38	3.4	1.5	Distress VAS: 39	3.6	1.5	Distress VAS: 34	1.8	1.4	Distress VAS: 34	3.7	1.6
Warth et al. 2020<br><br>**VAS from 0-10**	Distress VAS: 42	4.17	2.04	Distress VAS: 42	3.71	2.31	Distress VAS: 42	3.54	2.18	Distress VAS: 42	3.78	2.06
NG et al. 2016	Distress VAS: 30	6.29	1.66	Distress VAS: 30	6.00	1.77	Distress VAS: 30	4.71	1.57	Distress VAS: 30	5.79	2.01

*Values were either accessed directly from authors or computed by statistician from other variables
